# Association between shyness and depression among Chinese adolescents: the roles of social network site use and engagement in online games

**DOI:** 10.3389/fpsyg.2026.1814284

**Published:** 2026-05-20

**Authors:** Peipei Chen

**Affiliations:** Department of Marxism, Qingdao University of Science and Technology, Qingdao, China

**Keywords:** associated effect, depression, online games, shyness, social media site use

## Abstract

**Introduction:**

There is a substantial body of conceptual and empirical research discussing the relationship between shyness and depression, specific aspects of this relationship, such as the contribution of media use behaviors to the link between shyness and depression, remain underexplored and warrant further investigation. Thus, the present study investigated the associated effects of social network site use (SNSU) and engagement in online games (OG) on the association between shyness and depression among adolescents.

**Methods:**

Questionnaire responses from 5,215 Chinese adolescents were analyzed using structural equation modeling.

**Results:**

The following results were obtained: (1) shyness positively associated depression; (2) SNSU partially negatively associated the association between shyness and depression; and (3) engagement in OG partially positively associated the association between shyness and depression.

**Discussion:**

The findings suggest that different forms of media and patterns of consumption (e.g., SNSU versus engagement in OG) may differentially contribute to the association between shyness and depression. Therefore, the specific media context (e.g., potential strengthening versus compensatory effects) should be considered in future research and discussions.

## Introduction

Depression refers to a sad or unpleasant mood that an individual experiences over a period of time (Anne et al., 1993) and is more common in adolescents than other age groups (Balázs et al., 2013; Lu et al., 2024). In adolescents, depression can cause behavioral disorders (Lindner, 2017), eating disorders (Fewell et al., 2017), substance abuse (Lydecker et al., 2010), and suicidal tendencies (Balázs et al., 2013), underscoring its major effects on adolescents' physical and mental health. In this study, the mechanisms underlying the causes and development of adolescent depression were investigated to provide theoretical support for corresponding reduction measures.

Previous studies have demonstrated that individuals with shyness are prone to depression (Chan, 2012; Nudelman et al., 2024). Shyness is characterized by an uncomfortable, maladaptive state that emerges during interpersonal interactions, often accompanied by depression or inhibition (Henderson and Zimbardo, 2001). Shyness may affect depression through several routes (Joiner, 1997). First, individuals with shyness may have a negative self-perception and tend to overestimate others' expectations of them (Chan, 2012). In interpersonal communication, the discrepancy between high perceived expectations and low self-evaluation can cause individuals to experience internal conflicts and consequent unpleasant emotions, which in turn leads to depression (Kyte and Goodyer, 2008; Gao et al., 2020; Geng et al., 2021). Second, individuals with shyness tend to experience a high degree of social anxiety (Tsui et al., 2016) and often intentionally avoid certain social situations, causing them to miss opportunities to exercise their social ability (Rubin and Burgess, 2001). This behavior can cause an individual's social ability to degrade. These individuals may have difficulty coping with unavoidable social situations and thus experience negative emotions, which can lead to depression (Biggs et al., 2010).

Previous studies have indicated that individuals with shyness tend to use the Internet to seek psychological comfort and relieve negative emotions when facing real-life troubles (Tian et al., 2017). Although Internet use has some benefits for adolescents, it also causes numerous mental health problems (Banjanin et al., 2015). Existing studies have explored the association between Internet use and depression, revealing mixed results: whereas some studies have demonstrated a positive correlation between Internet use and depression (Lin et al., 2016), other studies have revealed a negative association (Morgan and Cotten, 2003) or no association (Jelenchick et al., 2013) between both variables. These inconsistent findings may suggest that different types of Internet activity have different effects on depression. Therefore, the associations between different types of Internet activity and depression require further investigation. Additionally, in most previous studies, Internet use has been included as an antecedent or outcome variable (Ko et al., 2014) of depression, but it has rarely been explored as a associated variable. In this study, SNSU and OG were examined as two specific types of Internet activity that may represent associated variables in the mechanism underlying the effect of shyness on depression.

### The role of SNSU

A social networking service (SNS) is a type of online platform that serves two main functions: to enable users to publish information and browse information published by others and to facilitate instant communication between users (Bae, 2017). In China, the two most widely used SNS tools are QQ and WeChat, which combine both functions. SNS are popular among adolescents and strongly associated with their mental health status (O'Keeffe and Clarke-Pearson, 2011; Yin et al., 2024). SNSU is defined as individuals use the SNS to present themselves, articulate their social networks, and establish or maintain connections with others (Ellison et al., 2007). Shyness affects both adolescents' in-person social behaviors and online social interactions (Daly, 2011). According to the theory of “the poor get poorer,” shy individuals with certain limitations in their social skills offline may also experience difficulties adapting during SNSU (Kraut et al., 2002), ultimately decreasing their SNSU. However, according to the social compensation model proposed by Kraut et al. (2002), key characteristics of SNSU, including anonymity, reality separation, and non-simultaneity (Jin, 2013), cause users experiencing shyness to feel less pressure when communicating on SNS, decreasing their shyness and strengthening their communication ability. The more comfortable social experience during SNS-based interaction, relative to in-person interaction (Stritzke et al., 2004), may increase SNSU among individuals with shyness. These inconsistent perspectives indicate that the association between shyness and SNSU requires verification through further research.

Depression has also been linked to SNSU. Frison and Eggermont (2015) reported that SNSU can reduce depression in adolescents. SNSU can satisfy multiple social needs of adolescents (Papacharissi and Rubin, 2000), promoting positive experiences that lower depression levels. Through SNSU, adolescents can also obtain social support, improve their self-esteem, and experience happiness, further reducing depression (Frison and Eggermont, 2015). However, several studies have indicated that SNSU can increase depression (e.g., Pantic et al., 2012), partially because teenagers frequently make social comparisons between their own abilities and opinions and those of others when coming across other people's version of their curated self (Lee, 2014), this version of oneself is often unrealistically polished (Reinecke and Trepte, 2014). When such information is used as the basis for comparison with oneself, individuals tend to think that others are better than them (Banjanin et al., 2015). This thought process generates non-adaptive cognition, further causing individuals to believe that they are not as good as others, and this low self-esteem can induce depression (Tandoc et al., 2015). In summary, although SNSU has been identified as a key factor affecting depression, its precise influence on depression requires further exploration. Additionally, shyness is highly correlated with SNSU, which in turn is highly correlated with depression. Thus, SNSU may associate the association between shyness and depression.

### The role of OG

Engagement in OG is another popular form of Internet use among adolescents. In moderation, playing OG has certain benefits for adolescent growth and development (Nurmagandi and Suratmini, 2024; Zhu et al., 2015), but this behavior may also adversely affect individuals. First, greater time spent playing OG reduces adolescents' study time, decreasing their learning engagement, which in turn can lead to lower grades, greater study pressure, and depression. Second, greater time spent playing OG reduces the time adolescents spend with family and friends, thereby reducing the quality of their interpersonal relationships and causing adolescents to experience unpleasant emotions that can increase depression (Mario and Martina, 2015). Additionally, some adolescents experiencing OG addiction may realize the negative effects of such behavior but have difficulty controlling themselves, reducing their sense of control and causing them to feel pessimistic and helpless, which increases depression (Kim and Ahn, 2016).

Shy individuals may be more likely to become addicted to OG (Tuncay, 2012) due to the personal characteristics of these individuals and the characteristics of the OG that they are playing. According to the ACE model proposed by Young (1997), key characteristics of OG include anonymity, convenience, and escapism. The escapist features of OG allow individuals encountering difficulties in life to temporarily forget their troubles through immersion in OG (Young, 1997). Engagement in OG can enable shy adolescents to temporarily escape from interpersonal problems and social anxiety in real life and obtain psychological comfort (Stetina et al., 2011). Additionally, shy adolescents frequently experience low self-esteem (Liao et al., 2016); accordingly, playing OG can satisfy their achievement (Stetina et al., 2011), autonomy (Ryan et al., 2006) and interpersonal (Ng and Wiemer-Hastings, 2005) needs, thereby improving their self-esteem. These findings suggest that shy adolescents engage more frequently in OG. Due to the high correlations between shyness and OG and between OG and depression, OG can be assumed to associate the association between shyness and depression.

### The present study

**Figure 1:**
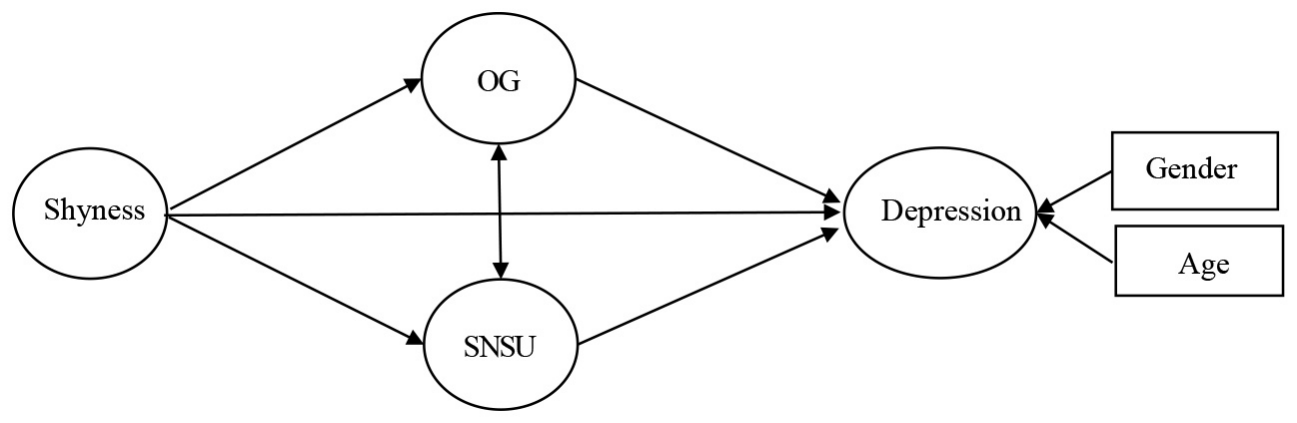
Theoretical model.

SNSU and OG may play different roles in the association between shyness and depression. This because shyness is characterized by social anxiety, social compensation model suggests SNSU could decrease their social anxiety and satisfy their social needs, which decrease the level of depression; however, engagement in OG would spend lager number of time of the individual with shyness even lead them to be addicted to it and bring them more negative emotions and the level of depression increased. Thus, such different roles should be tested. In order to further to test the association between shyness and depression, and the role of SNSU and engagement in OG, theoretical model is built (see Figure 1). We hypothesize (i) shyness could positively associate depression; (ii) shyness could negatively associate depression through SNSU; (iii) shyness could positively associate depression through engagement in OG.

## Research methods

### Participants

Based on the cluster sampling method, a total of 5,500 adolescents were selected from 4 schools in 3 cities in eastern China to complete a questionnaire survey. 285 questionnaires were excluded for excessive missing answers, answering too quickly and too slowly, all the same answers, a total of 5,215 valid questionnaires were collected. Among these participants, 2,303 were male (44.16%) and 2,827 were female (54.21%). The mean participant age was 16.19 years, and the standard error was 3.10 years. The present study was conducted in accordance with the 1964 Helsinki declaration and its later amendments or comparable ethical standards, with the approval of the Human Research Ethics Committee of Qingdao University of Science and Technology.

### Programs

All participating adolescents completed the questionnaire collectively during class. To ensure the quality of students' questionnaire responses, all students were evaluated by the class director (responsible for maintaining class order) and our researchers.

### Research tools

#### Shyness

The version of Henderson and Zimbardo's (2001) Shyness Scale revised by Wang et al. (2009) was used to measure participants' shyness. This scale consists of 17 items and is divided into four dimensions: approval seeking, self-blame, fear of rejection, and self-limitation of expression. Scale items are scored out of 5 points, with higher scores indicating a greater degree of shyness. In this study, the Cronbach's α coefficient of the scale was 0.865.

#### SNSU

The SNSU Scale was developed by Ellison et al. (2007), and scale items are scored out of 5 points with 6 items (for example, “QQ use and WeChat use are an integral part of my daily activities”). Higher scores indicate higher intensity of use. In this study, the Cronbach's α coefficient of the scale was 0.891.

#### OG

The OG Scale was developed by Király et al. (2015) and consists of 10 items that measure continuation, concentration, negative consequences, evasion, tolerance, loss of control, abandonment of other activities, cheating, and avoidance to assess the overall degree of engagement in OG. Scale items are scored out of 7 points, with higher total scores indicating greater engagement in OG. In this study, the Cronbach's α coefficient of the scale was 0.915.

#### Depression

The Center for Epidemiology Research Depression Scale was developed by Radloff (1977). The scale contains a total of 20 items that focus on the individual's emotional experience rather than the physical symptoms of depression. Scale items are scored out of 4 points, with higher total scores corresponding to more severe depression. In this study, the Cronbach's α coefficient of the scale was 0.892.

### Statistical methods

SPSS 18.0 was used for descriptive statistics and correlation analysis of the data. AMOS 22.0 was used for confirmatory factor analysis. MPLUS 7.0 software was used for structural equation model analysis and the examination of associated effects.

## Results

### Common method variance

Common method variance was assessed according to Harman's single factor method. Nine factors with eigenroots greater than 1 were identified, and the first factor explained 16.68% of the variance, which is below the threshold value of 40%. This finding indicates that common method bias was not a serious concern in the study data.

### Descriptive statistics, correlation analysis, and difference test

Table 1 lists the mean, standard deviation, and correlation matrix of each variable. The results of the correlation analysis revealed that shyness was positively correlated with SNSU, engagement in OG, and depression. Depression was positively correlated with SNSU and engagement in OG.

**Table 1:** Descriptive statistics and correlation analysis of study variables.

Variables	*M ±SD*	1	2	3	4	5	6
1. Gender	1.56 ± 0.52	1					
2. Age	16.20 ± 3.24	0.05^***^	1				
3. Shyness	46.30 ± 11.51	0.06^***^	0.07^***^	1			
4. Depression	17.71 ± 10.24	−0.03^**^	0.02	0.46^***^	1		
5. SNSU	35.89 ± 10.60	0.09^***^	0.36^***^	0.15^***^	−0.08^***^	1	
6. OG	24.43 ± 12.86	−0.03^***^	0.01	0.22^***^	0.34^***^	0.18^***^	1

^**^Correlation is significant at the 0.01 level (2-tailed).

^***^Correlation is significant at the 0.001 level (2-tailed).

### Structural equation model analysis

To control for the inflation measurement error caused by multiple latent variable items, the items measured by the OG and depression scales were grouped into two packages, and the total scores of the items contained in the two packages were then calculated and used to replace all model items. For the shyness and SNSU evaluations, subscale scores were used to represent total scores.

#### Measurement model test

MPLUS 7.0 was used to evaluate the degree of fit between the measurement model and the actual data. The results for the fit indices were as follows: χ^2^/*df* = 13.996, RMSEA = 0.050, CFI = 0.986, TLI = 0.978. These results indicate that the degree of model fit was ideal, and all observed variables fully and accurately reflected the latent variables; thus, the model was suited for further testing.

#### Structural equation model test

**Figure 2:**
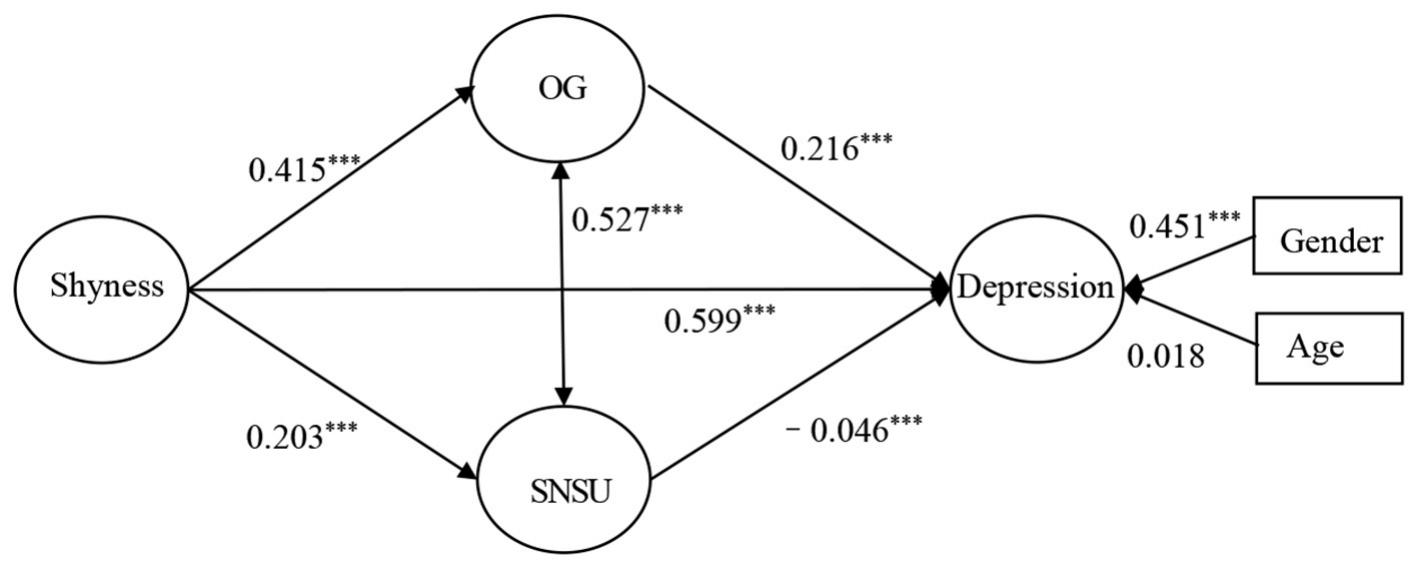
Associations between study variables. ***Correlation is significant at the 0.001 level (2-tailed).

The fit indices of the structural equation model were as follows; χ^2^/*df* = 10.408, RMSEA = 0.042, CFI = 0.986, TLI = 0.985. The model testing results are displayed in Figure 2. The path coefficients indicated that shyness significantly and positively associated depression, confirming hypothesis 1. Shyness also significantly and positively associated engagement in OG, and engagement in OG significantly and positively associated depression. Finally, shyness significantly and positively associated SNSU, and SNSU significantly and negatively associated depression.

#### The role of engagement in OG and SNSU

Engagement in OG significantly and positively associated the association between shyness and depression, as indicated by the absence of 0 in the confidence interval for the indirect effect from shyness to engagement in OG to depression (Table 2). SNSU significantly and negatively associated the association between shyness and depression, as indicated by the absence of 0 in the confidence interval for the indirect effect from shyness to SNSU to depression. These findings support hypothesis 2 and hypothesis 3.

**Table 2:** The role of OG and SNSU.

Paths	Effect	Bootstrap SE	Lower 95% CI	Upper 95% CI	Effect sizes
Shyness → OG → Depression	0.071 ^***^	0.006	0.068	0.106	0.128
Shyness → SNSU → Depression	−0.010^**^	0.004	−0.023	−0.002	0.013

^**^Correlation is significant at the 0.01 level (2-tailed).

^***^Correlation is significant at the 0.001 level (2-tailed).

## Discussion

### Association between shyness and depression

In this study, adolescents with greater shyness tended to experience more depression, confirming hypothesis 1. This finding is consistent with those of previous research (Chan, 2012). First, shy adolescents tend to pay particular attention to negative events in interpersonal communication (Chan, 2012) and often experience worries surrounding unavoidable social activities (Tsui et al., 2016), which can cause depression. Second, according to the cognitive theory of mood disorders proposed by Beck (1976), cognitive factors have a major effect on depression and other negative emotions. Similarly, Liu (2017) reported that shyness can increase individual depression through negative irrational cognition. Specifically, individuals experiencing shyness tend to evaluate and interpret events from a negative or pessimistic viewpoint and perceive themselves negatively, which can cause feelings of depression, helplessness, and inferiority and other negative emotions.

### The role of SNSU

This study demonstrated that SNSU partially associates the association between shyness and depression, suggesting that greater SNSU among shy adolescents reduces their degree of depression. This finding is consistent with those of previous studies (Frison and Eggermont, 2015) and supports the social compensation model (Kraut et al., 2002), which posits that shy individuals benefit from SNSU. The anonymity, reality separation, and non-simultaneity of Internet communication (Jin, 2013) greatly reduces the negative effect of low self-confidence and social skill limitations for shy adolescents who socialize online. This behavior enables these adolescents to experience satisfactory interpersonal relationships within a comfortable social atmosphere (Sheeks and Birchmeier, 2007). In turn, successful interpersonal communication reinforces adolescents' SNSU (Han et al., 2016).

Notably, the results of several previous studies are inconsistent with those of this study. For example, Niu et al. (2016) observed a positive correlation between SNSU and depression. This discrepancy may stem from differences in the definition of SNSU between both studies. Niu et al. measured SNSU among their study participants in terms of their engagement with the Qzone platform, focusing on the information release and browsing functions of SNS. They hypothesized that adolescents engage in social comparison when browsing information published by other SNS users and that such information is biased toward positivity (Qiu et al., 2012), decreasing the self-evaluation and self-esteem of adolescents who make such comparisons and thereby increasing depression. The present study focused on the QQ and WeChat platforms to facilitate examination of the instant messaging function of SNS, which better reflects the intensity of individual use of the chosen platforms to communicate socially with others. Through online socializing, individuals can establish and maintain social relationships with others and obtain greater social support (Frison and Eggermont, 2015), which reduces depression. This finding suggests that the specific functions of SNS must be differentiated in investigations of how SNSU affects an individual's mental health status. Although shy adolescents can be encouraged to communicate with others in moderation through SNS, they should also be guided to form a reasonable understanding of others' self-presentations on SNS to avoid depression induced by social comparison. Additionally, studies have found mindful use of social media, which was defined as users are aware of their environment, sensations, thoughts, and feelings during social media consumption, was helpful to improve metal health (Shabahang et al., 2024, 2026). From this point of view, mindful use of social media was suggested, which could make the SNSU more healthier.

### The role of engagement in OG

This study also demonstrated that engagement in OG partially associates the association between shyness and depression, suggesting that shy adolescents can become dependent on OG through increased time spent playing OG, which aggravates their depression. This result supports those of previous studies (Kim and Ahn, 2016).

Virtual reality and free play OG characteristics enable shy adolescents who experience negative emotions during real-world interpersonal communication to feel pleasure and satisfaction (Stetina et al., 2011), increasing their willingness to spend time playing OG. Moreover, positive in-game experiences reinforce individuals game-playing behavior, which can cause dependence on OG in the long term. Teachers and parents can somewhat reduce the intensity of adolescent engagement in OG through Internet supervision (Lai et al., 2014). However, shy adolescents with poor interpersonal relationships may lack communication and trust with teachers and parents or feel indifferent toward these relationships. In these cases, Internet supervision by parents and teachers may be both more difficult and less effective, causing shy adolescents to engage excessively in OG.

Excessive engagement in OG among adolescents can have adverse consequences, affecting adolescents' academic performance, increasing academic pressure, and eventually increasing depression (Zhu et al., 2015). When time spent playing OG replaces that spent socializing in real life with friends and relatives, interpersonal relationships may worsen, leading to a loss of social support and increasing susceptibility to negative events, thereby increasing depression (Mario and Martina, 2015). Additionally, adolescents may experience a decreased sense of control when they are aware of the adverse effects of their addiction to OG but are unable to change their behavior, especially shy adolescents, who often exhibit blame attribution tendencies. A lack of perceived control can cause adolescents to think of themselves negatively (Henderson and Zimbardo, 2001), which can increase depression.

### Limitations and future directions

Although the results of present study provided some new understandings of the association between shyness and depression and its mechanisms, several limitations existed. Firstly, self-reported survey was used, which may be influenced by common method bias. In order to solve it, the reported data from parents, teachers and classmates should be surveyed. Second, cross-sectional design was used, which made it hard to make a causal inference. Therefore, longitudinal and experimental design should be conducted to verify the results of present study. Thirdly, all the participants were Chinese students, which made it difficult to generalize the findings to other countries influenced by cultural differences. Thus, the results of present study should be verified with other country samples. Finally, due to the participants were selected from 4 schools in 3 cities in eastern China to complete a questionnaire survey, the the clustering of participants within schools/cities has not been modeled with the present study, and future study should solve this limitation.

## Conclusions

(1) Shyness is positive associated with depression;

(2) SNSU partial negative associates the association between shyness and depression;

(3) Engagement in OG partial associates the association between shyness and depression.

## Data Availability

The raw data supporting the conclusions of this article will be made available by the authors, without undue reservation.
